# Incidence and course of child malnutrition according to clinical or anthropometrical assessment: a longitudinal study from rural DR Congo

**DOI:** 10.1186/1471-2431-14-22

**Published:** 2014-01-28

**Authors:** Hallgeir Kismul, Catherine Schwinger, Meera Chhagan, Mala Mapatano, Jan Van den Broeck

**Affiliations:** 1 Centre for International Health, University of Bergen, 5020 Bergen Norway; 2 Department of Paediatrics, University of KwaZulu-Natal, 4013 Congella, South Africa; 3 School of Public Health, University of Kinshasa, Kinshasa 1, Democratic Republic of Congo

**Keywords:** Malnutrition, Marasmus, Kwashiorkor, Wasting, Stunting, Incidence

## Abstract

**Background:**

Longitudinal studies describing incidence and natural course of malnutrition are scarce. Studies defining malnutrition clinically [moderate clinical malnutrition (McM) marasmus, kwashiorkor] rather than anthropometrically are rare. Our aim was to address incidence and course of malnutrition among pre-schoolers and to compare patterns and course of clinically and anthropometrically defined malnutrition.

**Methods:**

Using a historical, longitudinal study from Bwamanda, DR Congo, we studied incidence of clinical versus anthropometrical malnutrition in 5 657 preschool children followed 3-monthly during 15 months.

**Results:**

Incidence rates were highest in the rainy season for all indices except McM. Incidence rates of McM and marasmus tended to be higher for boys than for girls in the dry season. Malnutrition rates increased from the 0–5 to the 6 – 11 months age category. McM and marasmus had in general a higher incidence at all ages than their anthropometrical counterparts, moderate and severe wasting. Shifts back to normal nutritional status within 3 months were more frequent for clinical than for anthropometrical malnutrition (62.2-80.3% compared to 3.4-66.4.5%). Only a minority of moderately stunted (30.9%) and severely stunted children (3.4%) shifted back to normal status. Alteration from severe to mild malnutrition was more characteristic for anthropometrically than for clinically defined malnutrition.

**Conclusions:**

Our data on age distribution of incidence and course of malnutrition underline the importance of early life intervention to ward off malnutrition. In principle, looking at incidence may yield different findings from those obtained by looking at prevalence, since incidence and prevalence differ approximately differ by a factor “duration”. Our findings show the occurrence dynamics of general malnutrition, demonstrating that patterns can differ according to nutritional assessment method. They suggest the importance of applying a mix of clinical and anthropometric methods for assessing malnutrition instead of just one method. Functional validity of characterization of aspects of individual nutritional status by single anthropometric scores or by simple clinical classification remain issues for further investigation.

## Background

While the worldwide prevalence of child malnutrition in the period from 1990 to 2010 declined significantly, there has been only minimal change in sub-Saharan Africa [1]. It is therefore important to improve our understanding of child malnutrition in these settings. Many studies from sub-Saharan Africa have determined the national, regional or local occurrence frequencies of child malnutrition. Typically, these studies provide prevalence rates of low anthropometric scores in population cross-sections as the measure of burden of malnutrition. In contrast, longitudinal studies looking at incidence and natural course of malnutrition are few. Such studies are useful because they allow for a better understanding of season- and age- dependent risks for developing malnutrition. The study of the natural course of malnutrition is considered to be of particular value for nutritional programmes in planning interventions [2]. There are very few such studies and according to Isanaka et al. [3] only one population-based study has been published concerning the duration of untreated malnutrition [4]. Studies defining malnutrition clinically (marasmus, kwashiorkor, moderate clinical malnutrition) rather than anthropometrically are also scarce, despite the fact that anthropometric assessment alone lacks specificity in the diagnosis of malnutrition [5].

Given that clinical assessment of malnutrition is a comparatively inexpensive method suitable for regions with a significant burden of malnutrition, the lack of attention to this method is remarkable.

The aim of this paper is to address, in a large population-based study, longitudinal occurrence patterns and course of malnutrition among pre-schoolers and to compare these patterns among clinically and anthropometrically defined malnutrition. Our specific aim was to describe age-, season- and gender- dependent incidence of moderate clinical malnutrition, marasmus and kwashiorkor, and compare these with rates obtained using anthropometrical definitions of malnutrition. We also sought to describe and compare patterns of change and duration of clinically and anthropometrically defined malnutrition.

## Methods

### The Bwamanda study

This paper presents a secondary analysis of data from the historical Bwamanda study [6]. The rural area of Bwamanda is located in northwest DR Congo and has a tropical climate with the rainy season lasting from April to November and the dry season from December to March. The major livelihood adaptation was subsistence agriculture, mainly cultivation of cassava and maize. The area was served by a central hospital and 10 peripheral health centres with a local NGO that up till today holds the major responsibilities for running the health services in the area. Several health centres had an associated nutritional rehabilitation centre, but the uptake was limited due to time constraints of mothers, the voluntary nature of the personnel services in these centres, and interruptions of stocks of food supplements. During the study sick children were referred to the local health centre or hospital where they received oral rehydration therapy for diarrhoea, antibiotics for severe respiratory infection and chloroquine or quinine for malaria. Moreover, severely malnourished children were offered transport to the Bwamanda hospital. Since the study was undertaken there have been few political and economic changes. The socio-economic development in the area has been constrained by several factors including restricted public service support and only minor private sector growth.

The study included 5 657 children from 16 villages in the Bwamanda area. A sample of 4 238 pre-school children was enrolled at the first contact. During follow-up newborn and immigrated children were added, while some children were lost due to emigration or death. In the last follow up round children who were born in 1984, and had reached six years, were no longer examined. Children were followed in the period 1989–1991. Three-monthly contacts were organised making up 15 months of follow-up and 6 contacts. The area was very homogeneous and there were no significant differences between the villages in nutritional status of the children or socioeconomic status (negligible design effect).

Fifteen interviewers holding a secondary school certificate were trained in simple physical examinations and in undertaking interviews in the villages according to an interviewer’s manual. They determined age on the basis of children’s birth date noted on road to health charts or/and on parents’ identity papers. This information was available for about 90% of the children. For the remaining ones, birth dates were determined by a careful interview of the mothers using a local events calendar.

Nutritional status of children was assessed by clinical assessment as well as by anthropometrical assessment. The clinical assessment of nutritional status is described by Van den Broeck et al. [7]. With this method marasmus was assessed by inspection of abnormal visibility of skeletal structures and by absence or near-absence of palpable gluteus muscle. Kwashiorkor was assessed using the presence of pitting oedema of the ankles and/or feet as a criterion. Moderate clinical malnutrition (McM) was identified as the presence of wasting of the gluteus muscle, wasting at inspection and/or palpation without signs of marasmus or kwashiorkor. Length of children below 12 months was measured with a locally constructed length measuring board, while older children’s standing height was measured with a microtoise, in both cases to the nearest 0.1 cm. A spring scale (CMS weighting equipment) was used to weigh the children to the nearest 100 gram. For the present analysis, anthropometric scoring was done using the WHO-MGRS 2006 Child Growth Standards [8]. Z-scores were calculated for weight for length/height (WHZ) and for length/height for age (HAZ). Children with a WHZ <-2 to >-3 were classified as moderately wasted, those with WHZ <-3 as severely wasted. Similarly, those with a HAZ <-2 to >-3 were categorised moderately stunted and those with HAZ <-3 as severely stunted. Clinical and anthropometric assessments partly take into account different aspects of malnutrition. Both clinical and anthropometric assessments are able to capture wasting processes and are therefore directly comparable methods. However, only anthropometric assessment measures stunting processes.

### Incidence rates of malnutrition

Incidence rates of the various forms of clinical and anthropometrical malnutrition were calculated for the age categories 0–5, 6–11, 12–23, 24–35 and 36–71 months. Incident cases were defined as malnutrition being present, but absent at the scheduled previous contact. For the calculation of incidence rates, the person-time at risk was defined on the basis of time elapsed from one contact round to the next, normally about 3 months. Incidence rate was expressed as number per 1 000 person months. Direct age standardization was used to compare incidence rates across seasons by using the age distribution in the first follow up round (second contact) as the reference. Season was defined as: dry post-harvest (January – March); beginning of rainy pre-harvest (April – June); rainy (July – September); end of rainy season post-harvest (October –December).

### Natural course of incident malnutrition

To document the natural course of incident malnutrition we examined short-term (3-months) shifts in severity, and short-term (3-months) mortality among children with incident malnutrition. Duration was categorised as 0–3, 3–6, 6–9, 9–12 months, or as censored after end of follow-up. Children with a WHZ and HAZ higher than <-2 were classified as normal, that is “no wasting” and “no stunting”.

### Ethical aspects

Ethical approval for the Bwamanda study had been granted by the University of Leuven’s Tropical Childcare Health Working Group and funding provided by the Flemish Inter-University Council and the Nutricia Research Foundation.

## Results

### Seasonal, gender and age distribution of malnutrition incidence

**Figure 1:**
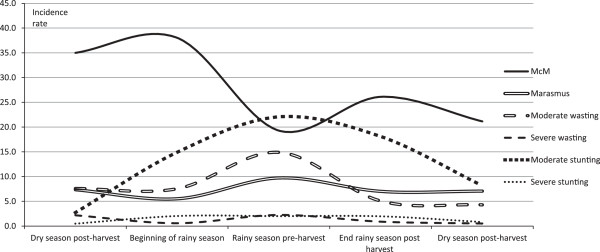
**Seasonality of malnutrition for incidence rates of moderate clinical malnutrition (McM), marasmus, moderate wasting, severe wasting moderate stunting and severe stunting).** The incidence rates are given per 1 000 child months. n = 3 620. The numbers for occurrence of kwashiorkor were comparatively too low to be presented. Age is given in months.

Figure 1 shows that incidence rates of marasmus and anthropometric malnutrition were lowest in the dry season and became highest in the rainy season. The incidence rates of McM were highest in the dry season. The rates declined in the middle of the rainy season but increased again at the end of the rainy season. The incidence rates of wasting were particularly high in the rainy season. The rates for moderate stunting were low in the dry season and highest in the rainy season. Severe stunting was low during the dry season and high from the beginning of rainy season to up to the dry season post- harvest. The incidence rate for kwashiorkor was highest in the end of the early rainy season with an incident rate of 1.4 per 1 000 child-months (not shown in figure).

As shown in Table 1, gender differences in incidence of malnutrition varied according to type and severity of malnutrition and according to assessment method. In all seasons there was a tendency for the incidence rate of McM to be higher in boys than in girls, but only significantly higher in the dry season post-harvest [for boys 41.3 4 per 1 000 child-months (95% CI: 35.4, 48.2) vs. for girls 28.74 per 1 000 child-months (95% CI: 23.8, 34.7)]. In the dry season the incidence rate of marasmus was also significantly higher for boys than for girls [12.0 per 1 000 child-months (95% CI: 9.0, 16.1) in boys 3.5 per 1 000 child-months (95% CI: 2.0, 6.0) in girls]. For anthropometrically defined malnutrition, there was no significant gender inequality in incidence of malnutrition, except for a higher incidence of moderate stunting in girls than in boys in the end of the rainy season, post-harvest [for girls 22.2 per 1 000 child-months (95% CI: 19.0, 26.0) vs. for boys 15.6 per 1 000 child-months (95% CI: 13.0, 18.7)].

**Table 1:** Incidence rate by gender and seasons of moderate clinical malnutrition (McM), marasmus, moderate wasting, severe wasting, moderate stunting and severe stunting

	**Age standardized incidence rate per 1 000 child-month, (95% CI)**							
	**Dry season, post-harvest**		**Beginning rain, pre-harvest**		**Rainy season**		**End of rainy season, post-harvest**	
	***Girls***	***Boys***	***Girls***	***Boys***	***Girls***	***Boys***	***Girls***	***Boys***
**Clinical malnutrition**								
McM^1^	28.7 (23.8, 34.7)	41.3 (35.4, 48.2)*	35.5 (30.1, 41.8)	42.3 (36.3, 49.2)	16.1 (13.1, 19.8)	22.2 (18.7, 26.3)	23.0 (19.8, 26.6)	29.5 (25.9, 33.6)
Marasmus^2^	3.5 (2.0, 6.0)	12.0 (9.0, 16.1)*	3.5 (2.1, 5.9)	7.3 (4.4, 12.3)	9.7 (7.3, 12.8)	8.5 (6.4, 11.4)	6.1 (4.6, 8.1)	7.1 (5.5, 9.2)
Kwashiorkor^3^	0.5	0.0	0	0.2	0.7 (0.3, 1.9)	1.7 (0.9, 3.2)	1.7 (1.0, 2.9)	0.8 (0.4, 1.7)
**Anthropometrical malnutrition**								
Moderate wasting^4^	7.9 (5.4, 11.4)	5.6 (3.7, 8.6)	6.5 (4.4, 9.6)	9.3 (6.8, 12.8)	9.9 (7.5, 13.1)	14.8 (11.9, 18.5)	5.7 (4.2, 7.6)	6.9 (5.4, 9.0)
Severe wasting^5^	0.3 (0.0, 2.0)	1.6 (0.7, 3.6)	0.4 (0.1, 1.9)	0.6 (0.2, 2.2)	1.5 (0.7, 3.0)	3.1 (1.9, 5.1)	1.0 (0.5, 2.0)	1.1 (0.5, 1.9)
Moderate stunting^6^	32.8 (27.2, 39.7)	27.8 (22.7, 34.0)	16.6 (12.9, 21.4)	13.5 (10.3, 17.7)	22.9 (18.9, 27.7)	21.9 (18.1, 26.5)	22.2 (19.0, 26.0)	15.6 (13.0, 18.7)*
Severe stunting^7^	2.1 (1.0, 4.5)	3.5 (2.0, 6.2)	2.3 (1.2, 4.5)	2.1 (1.0, 4.2)	1.2 (0.5, 3.0)	2.7 (1.6, 4.7)	1.2 (0.6, 2.4)	2.7 (1.8, 4.2)

The incidence rates are given per 1 000 child months. N = 3 620. *Confidence interval non-overlapping with that of girls.

^1^Identified as the presence of wasting of the gluteus muscle at inspection and/or palpation without signs of marasmus or kwashiorkor.

^2^Assessed by inspection of abnormal visibility of skeletal structures and by absence or near-absence of palpable gluteus muscle.

^3^Assessed using the presence of pitting oedema of the ankles and/or feet as a criterion.

^4^Weight-for-length/height Z-score <-2 to >-3.

^5^Weight-for-length/height Z-score <-3.

^6^Length/height-for-age Z-score <-2 to >-3.

**Figure 2:**
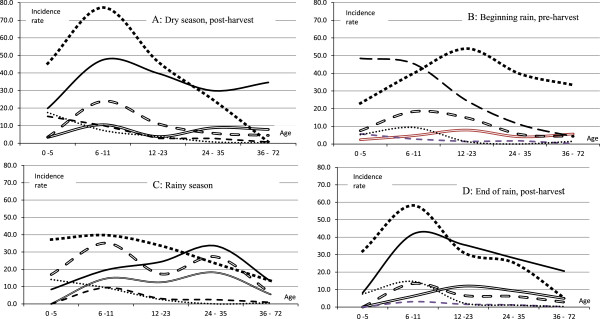
**Incidence rates according to age and stratified by season of moderate clinical malnutrition (McM), marasmus, moderate wasting severe wasting, moderate stunting and severe stunting.** The incidence rates are given per 1 000 child months. n = 3 620. The numbers for occurrence of kwashiorkor were comparatively too low to be presented. Age is given in months. Standards [8]. The incidence rates are given per 1 000 child months. n = 3 620. The numbers for occurrence of kwashiorkor were comparatively too low to be presented. Age is given in months.

Figure 2 shows that the incidence rates of malnutrition increased from the 0–5 to the 6 – 11 months age categories in all seasons. In the 3 older age categories (12 – 23, 24 – 35 and 36–72 months) the rates tended to decline with increasing age, also in all seasons. During the rainy season (Panel C) the age-dependent decrease in incidence of MCM, moderate wasting and marasmus appears 'delayed’ until after the age of 36 months. In general, clinical malnutrition (McM and marasmus) had a higher incidence at all ages than their anthropometrical counterpart (moderate and severe wasting). The rates for moderate stunting were higher than any other forms of malnutrition up to the age of 12 months. While moderate stunting incidence is very high at younger ages, it becomes lower at older ages. Severe stunting shows a similar pattern, namely an increase up to the age of 23 months and a decrease after that.

Kwashiorkor was the least frequent type of malnutrition (not shown in Figure 2), with the highest incident rate (2.9 per 1 000 person months) in the rainy season for the age category 24–35 months.

### Natural course of incident malnutrition

Table 2 shows that the proportions shifting (3-months shifts) from one level or severity of malnutrition to another differed between clinically malnourished and anthropometrically malnourished children. The percentage of children shifting back to a normal nutritional status within 3 month was higher for clinical malnutrition than for anthropometrical malnutrition (62.2-80.3% compared to 3.4-66.4%). The majority of incident cases normalised after three months, except for stunting where only a minority normalised from moderate (30.9%) or severe stunting (3.4%).

**Table 2:** Shifts in severity of malnutrition after 3 months in children with incident of moderate clinical malnutrition (McM), marasmus, moderate wasting, severe wasting moderate stunting and severe stunting

	**Total number of incident cases**	**Nutritional status after three months % (95% CI)**			
		**McM**	**Marasmus**	**Kwashiorkor**	**Normal clinical status**
McM^1^	1044	20.4 (18.0, 22.8)	2.5 (1.6, 3.4)	0,3	74.3 (71.3, 76.7)
Marasmus^2^	198	5.1 (2.0, 8.2)	9.6 (5.5, 13.7)	1.0	80.3 (74.4, 85.6)
Kwashiorkor^3^	37	0	0	24.3 (10.5, 38.1)	62.2 (44.6, 77.8)
		**Moderate wasting**	**Severe wasting**		**No wasting**
Moderate wasting^4^	232	25.0 (19.4, 30.6)	4.7 (2.0, 7.4)		66.4 (60.3, 72.5)
Severe wasting^5^	61	27.9 (16.6, 39.2)	11.5 (3.5, 19.5)		52.5 (40.0, 65.0)
		**Moderate stunting**	**Severe stunting**		**No stunting**
Moderate stunting^6^	687	57.2 (53.5, 60.9)	7.7 (5.7, 9.7)		30.9 (27.4, 34.4)
Severe stunting^7^	557	32.1 (28.2, 36.0)	62.5 (58.5, 66.5)		3.4 (1.9, 4.9)

^1^Identified as the presence of wasting of the gluteus muscle at inspection and/or palpation without signs of marasmus or kwashiorkor.

^2^Assessed by inspection of abnormal visibility of skeletal structures and by absence or near-absence of palpable gluteus muscle.

^3^Assessed using the presence of pitting oedema of the ankles and/or feet as a criterion.

^4^Weight-for-length/height Z-score <-2 to >-3.

^5^Weight-for-length/height Z-score <-3.

^6^Length/height-for-age Z-score <-2 to >-3.

^7^Length/height-for-age Z-score <-3.

Nutritional status more often remained unchanged in children with moderate forms of wasting (McM and moderate wasting) than in children with severe (severe marasmus and severe wasting) forms of wasting (20.4-25% compared to 9.6-11.5%). As to incident kwashiorkor, 24.3% still presented with kwashiorkor the following round. For stunting, as many as 57.2% of those with moderate forms and 62.5% of those with severe forms had not shifted after 3 months. Alteration from severe to mild forms was more characteristic for anthropometrical than for clinical malnutrition, with the percentage for severe wasting and severe stunting being 27% and 32.1%.

Table 3 describes duration of moderate forms of malnutrition according to season of start of the malnutrition episode. There were no significant differences between McM and moderate wasting. The percentage of McM resolving after 3 months was 64.4% to 76.7% depending on the season, and for moderate wasting 69.2% to 78.3%. Children with moderate stunting resolving after 3 months were a minority (18.4% to 35.3%). A large percentage of children with moderate stunting remained stunted even after 9 to 12 months.

**Table 3:** Duration of incident moderate clinical malnutrition (McM), moderate wasting and moderate stunting

**Season at start of malnutrition**	**Total number of incident cases in the season**	**Return to normal nutritional status:**			
		**After 3 months % (95% CI)**	**After 6 months (%, 95% CI)**	**After 9 months (%, 95% CI)**	**After 12 months (%, 95% CI)**
*McM*^*1*^					
Dry season, post- harvest	100	76.0 (67.6, 84.4)	19.0 (12.6, 24.2)	2.2	2.0
Dry season, pre-harvest	159	76.7 (70.1, 83.3)	8.2 (3.9, 12.5)	8.8 (4.4, 13.2)	Censored
Rainy season, pre-harvest	174	64.4 (57.3, 71.5)	18.4 (12.6, 24.2)	Censored	
End of rainy season, post-harvest	317	71.0 (66.0, 76.0)	Censored		
*Moderate wasting*^*2*^					
Dry season, post- harvest	26	69.2 (51.5, 86.9)	15.4 (1.5, 29.3)	3.8	0.0
Dry season, pre-harvest	27	70.4 (53.2, 87.6)	22.2 (6.5, 37.9)	No cases	Censored
Rainy season, pre-harvest	60	78.3 (67.9, 88.7)	15.0 (6.0, 24.0)	Censored	
End of rainy season, post-harvest	66	75.8 (65.5, 86.1)	Censored		
*Moderate stunting*^*3*^					
Dry season, post- harvest	68	35.3 (23.9, 46.7)	8.8 (2.1, 15.5)	4.4	10.3 (3.1, 17.5)
Dry season, pre-harvest	52	23.1 (11.6, 34.6)	5.8	13.5 (4.2, 22.5)	Censored
Rainy season, pre-harvest	147	18.4 (12.1, 24.7)	19.0 (12.7, 25.3)	Censored	
End of rainy season, post-harvest	231	32.0 (26.0, 38.0)	Censored		

^1^Identified as the presence of wasting of the gluteus muscle at inspection and/or palpation without signs of marasmus or kwashiorkor.

^2^Weight-for-length/height Z-score <-2 to >-3.

^3^Length/height-for-age Z-score <-2 to >-3.

## Discussion

Earlier studies on malnutrition among preschool children have primarily provided prevalence rates of low anthropometric scores in population cross-sections. To our knowledge the current study is among the first to provide incidence rates according to basic determinants and season, and to compare incidence rates of clinically and anthropometrically defined malnutrition. We have shown that seasonal, gender and age distribution as well as course of malnutrition are different when defining malnutrition clinically instead of anthropometrically. For example, we have shown that clinical forms of malnutrition had in general higher incidence rates than their anthropometric counterparts.

The people of Bwamanda are predominantly subsistence farmers and availability of food is strongly influenced by seasonal climatic changes. Our study largely confirmed the findings of other studies showing that the risk of developing malnutrition is especially high in the rainy season [9-11]. We speculate that the high incidence of wasting and stunting in the rainy season could relate to increased morbidity from diarrhoea and malaria whereas the high incidence of McM at the end of the dry season may rather reflect changes in food access depending on the cropping season. Local farmers typically face food shortage during the dry season with a notable shortage prior to the first harvesting of maize in mid-June. However, if we consider age distribution, we found that for the 24–35 months age range the incidence rate of McM was also high in the rainy season.

We found significant gender inequality in the incidence of McM and Marasmus, with the incidence rate being higher for boys than for girls in the dry season. For other forms, both clinically and anthropometrically defined, we did not find that incidence of malnutrition was higher in boys than in girls. However, in one season we found that the incidence of moderate stunting was higher in girls than in boys. There are other studies that have found associations of gender with malnutrition. For example, in a study using data from 16 DHS (Demographic and Health Surveys) in 10 sub-Saharan countries, Wamani et al. found that boys were more frequently stunted than girls [12]. In comparison, in our study incidence of stunting showed no gender difference. Using nine WFS (World Fertility Surveys) and 51 DHS surveys undertaken in Sub-Saharan Africa Garenne et al. examined prevalence of malnutrition and found prevalence of underweight (low weight-for-age) to be higher among boys than girls [13]. We did not examine low weight for age but found that there was no gender difference in incidence of low weight for length/height.

Our study demonstrates that malnutrition incidence at different ages varied according to clinically and anthropometrically defined malnutrition. Still the general pattern for all forms of malnutrition was that incidence was higher at ages 6–36 months than before or after. In a cross sectional study from Uganda Kikafunda et al. found that the risk of older children being stunted relative to younger children were 6 times higher for those in the 12–18 month age range and 10 times higher in the age group above 18 months [14]. While Kikafunda et al. studied prevalence rates, we studied incident rates and found that the risk of developing stunting is high at ages below 12 months and declines at the 12–23 months age range. Our study therefore supports recent studies emphasising the sensitivity of linear growth to environmental factors during the child’s early two years of life [15]. In line with this Victora et al. and Miamady et al., analysing WHO national anthropometric data from 54 countries and Indian National Family Health Survey respectively, found that mean HAZ declined dramatically until at the age of 24 months [16,17]. In Bwamanda weaning food is already introduced at the age of 3 months and this early introduction could explain the high incidence rates of malnutrition in infancy.

We have described the frequency of severity shifts and returns to normal nutritional status after three months. The percentage of children with marasmus or McM who returned to normal was high. It was also noticeable that a large proportion of severely stunted children returned to moderate stunting. Isanaka et al. estimated the duration of untreated acute moderate and severe anthropometrical malnutrition, defined by WHZ and absolute MUAC (mid-upper arm circumference), by a mathematical model and data from a community-based cohort in Niger of children aged 6 to 60 months [3]. Using the 2006 World Health Organization growth standards their study estimated the duration of moderate acute malnutrition to be 2.5-2.7 months (WHZ defined) and 3.4 – 3.9 months (MUAC defined). Isanaka et al. estimated the duration of severe acute malnutrition at 1.5 months (WHZ defined). In our study most of the incident cases of McM and moderate wasting resolved after 0–3 months which suggests that the duration of episodes were more in accordance with the study of Isanaka et al. with regards to moderate malnutrition. The suggested duration of malnutrition was thereby shorter than the duration found in an earlier study by Garrenne et al. [4]. The latter study estimated severe malnutrition (severe wasting) to last 7–8 months on average. We did not have sufficient incident cases in our study to estimate the duration of severe malnutrition with useful precision. Since caretakers were offered assistance this might have influenced the duration of episodes of malnutrition in our study.

Our analysis was based on a large sample of pre-school children, but a weakness is that many children were lost due to emigration and during follow up. This weakness in particular constrained our examination of the duration of malnutrition for severe malnutrition. In order to understand how emigration and lost to follow up might have influenced our findings we compared last nutritional status of children who emigrated or were lost to follow up with children who also were surveyed in the subsequent follow up round. This analysis yielded no evidence that emigration and lost to follow up influenced our findings. Data on incidence and course of malnutrition were obtained from two sequential follow up rounds and thereby dependent on two different measurements. The data on incidence and course of malnutrition were thereby susceptible to measurement errors. We are also aware that we might not have captured some of the shorter episodes of malnutrition which occurred and were resolved between visits.

## Conclusions

Our data on age distribution of incidence of malnutrition underlines the importance of strengthening interventions before children reaches the age of 2 years to ward off malnutrition. Our findings, especially with regard to course of McM, marasmus and severely stunted children, emphasise the importance of early life intervention.

There are few population-based studies that have addressed the occurrence dynamics of clinically and anthropometrically defined malnutrition. Our findings show the occurrence dynamics of general malnutrition in a rural African area, demonstrating that patterns can differ according to nutritional assessment method. None of the assessment methods can be described as superior as they partly measure different aspects of malnutrition. Our findings suggest the importance of applying a mix of clinical and anthropometric methods for assessing malnutrition instead of just one method. Functional validity of aspects of characterization of individual nutritional status by single anthropometric scores or simple clinical classifications remain issues for further investigation.

## Abbreviations

HAZ: Length/height for age Z-score; McM: Moderate clinical malnutrition; NGO: Non-governmental organisation; WHZ: Weight for length/height Z-score.

## Competing interests

The authors declare that they have no competing interests.

## Authors’ contributions

HK, CS, MC, MMA, JVdB participated in the conception of the study. H.K. performed data analysis and wrote the paper. All authors participated in the revision of the paper. All authors read and approved the final manuscript.

## Pre-publication history

The pre-publication history for this paper can be accessed here:

http://www.biomedcentral.com/1471-2431/14/22/prepub

## Accessibility of the Bwamanda dataset

As the principle investigator Jan Van den Broeck is the custodian of the Bwamanda dataset. Jan Van den Broeck supervised our study and provided Hallgeir Kismul as the first author access to the Bwamanda data. The dataset can be made available by contacting Jan Van den Broeck; Jan.Broeck@cih.uib.no.
